# Offering Fiber-Enriched Foods Increases Fiber Intake in Adults With or Without Cardiometabolic Risk: A Randomized Controlled Trial

**DOI:** 10.3389/fnut.2022.816299

**Published:** 2022-02-16

**Authors:** Beate Brandl, Rachel Rennekamp, Sandra Reitmeier, Katarzyna Pietrynik, Sebastian Dirndorfer, Dirk Haller, Thomas Hofmann, Thomas Skurk, Hans Hauner

**Affiliations:** ^1^ZIEL-Institute for Food and Health, Technical University of Munich, Freising, Germany; ^2^Chair of Nutritional Medicine, Else Kroener-Fresenius-Centre for Nutritional Medicine, TUM School of Life Sciences, Technical University of Munich, Freising, Germany; ^3^Chair of Food Chemistry and Molecular Sensory Science, TUM School of Life Sciences, Technical University of Munich, Freising, Germany; ^4^Chair of Nutrition and Immunology, Technical University of Munich, Freising, Germany; ^5^Institute of Nutritional Medicine, School of Medicine, Technical University of Munich, Munich, Germany

**Keywords:** fiber-enriched foods, fiber, patient satisfaction, healthy diet, microbiome

## Abstract

**Introduction:**

Previous efforts to increase fiber intake in the general population were disappointing despite growing awareness of the multiple benefits of a high fiber intake. Aim of the study was to investigate the acceptance and consumption of fiber-enriched foods.

**Methods:**

One hundred and fifteen middle-aged healthy individuals with and without elevated waist circumference (> 102 cm in males and > 88 cm in females) were recruited and randomized to an intervention or an age- and sex-matched control group. Subjects assigned to the intervention group were invited to select fiber-enriched foods from a broad portfolio of products to increase fiber intake by 10 g/day. Control subjects could choose items from the same food basket without fiber enrichment. The primary outcome was the increase in dietary fiber intake, and secondary outcomes were changes in cardiometabolic risk factors, microbiota composition, food choices, and consumer acceptance of the fiber-enriched foods.

**Results:**

Compared to baseline, daily fiber intake increased from 22.5 ± 8.0 to 34.0 ± 9.6 g/day after 4 weeks (*p* < 0.001) and to 36.0 ± 8.9 g/day after 12 weeks (*p* < 0.001) in the intervention group, whereas fiber intake remained unchanged in the control group. Participants rated the taste of the food products as pleasant without group differences. In both groups, the most liked foods included popular convenience foods such as pretzel breadstick, pizza salami, and pizza vegetarian. After 12 weeks of intervention, there were minor improvements in plasma lipids and parameters of glucose metabolism in both the intervention and control group compared to baseline, but no differences between the two groups. Increased fiber consumption resulted in an increased (*p* < 0.001) relative abundance of *Tannerellaceae*.

**Conclusions:**

Fiber-enrichment of popular foods increases fiber intake in a middle-aged population with and without cardiometabolic risk and may provide a simple, novel strategy to increase fiber intake in the population.

## Introduction

A high fiber and whole-grain intake is regularly recommended to the population to prevent or reduce cardiometabolic and other diet-related diseases (1–3). Especially, diets high in dietary fiber are known to have protective effects against obesity, type 2 diabetes, cardiovascular disease, and some types of cancer (4–8). These recommendations are mainly based on observational studies, but also supported by a growing number of usually small and short-term randomized controlled studies (RCTs) on the beneficial effects of fiber-rich/whole-grain foods on diet-related diseases (9, 10).

Although there is substantial heterogeneity across trials, including variability in study design, duration, types of fiber products, significant improvements of risk factors including fasting and postprandial plasma glucose and insulin, total and LDL-cholesterol were reported (11, 12). Furthermore, whole-grain/fiber-rich foods are a good source of vitamins, minerals, lignans, and other phytochemicals and, therefore, may provide additional health benefits (13). A comprehensive review of the literature recently demonstrated a 15–30% decrease in all-cause mortality and morbidity from cardiovascular disease, type 2 diabetes, and colorectal cancer when comparing a high with a low dietary fiber intake in adults (14).

Similar low dietary fiber intake has been reported from European countries and North America (15). Although the public is aware of the benefits of fiber, the majority of Americans and Europeans fall exceedingly short of meeting the recommendations from nutrition experts and societies. This gap in fiber intake was also observed in Germany. The second German National Nutrition surveys (NVS II) reported that the current median intake of dietary fiber in the adult German population is 23 g/d in females and 25 g/d in males, markedly below the recommended intake level of > 30 g/day of the German Nutrition Society (DGE) (16, 17). Therefore, an increase in fiber intake in the general population is strongly promoted by most authorities. Despite this clear message in public health activities, consumer's acceptance of fiber-rich foods is low, while the sensory perception of fiber-rich foods by consumers remains elusive. To date, there is little known about consumer preference and acceptance of fiber-enriched foods.

To address this topic, we were interested in studying if provision of popular fiber-enriched foods may increase dietary fiber intake and how consumers accept and rate various fiber-enriched food groups. We were also interested in seeing if a potentially higher fiber consumption via offering fiber-enriched foods may affect cardiometabolic health and gut microbiome composition.

## Subject and Methods

### Ethic Statement

The study protocol was approved by the Ethical Committee of the Faculty of Medicine of the Technical University of Munich (Approval no. 201/17S). All procedures were in agreement with the Declaration of Helsinki of 1975, as revised in 2013. Written informed consent was obtained from all participants before enrollment. The protocol was registered in the German Clinical Trials Register (DRKS00011528).

### Study Participants

**Figure 1:**
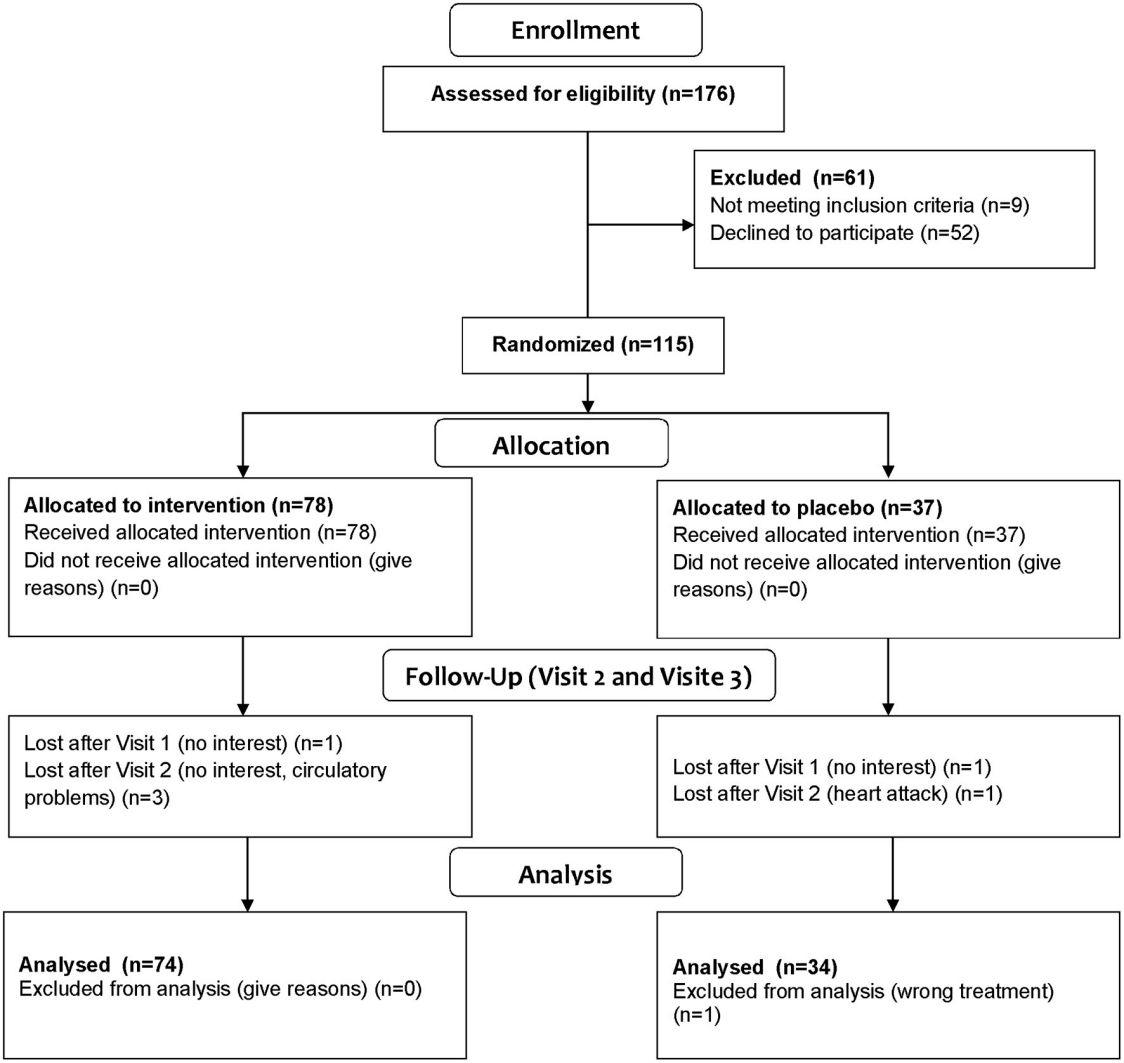
CONSORT flow chart of study participants and intervention.

Individuals aged 40–65 years were initially recruited and phenotyped within the *enable* cluster of nutrition research (18), and were invited to participate in this so-called Freising Fiber Acceptance study (Figure 1) to increase fiber intake by offering fiber-enriched convenience food products from various food groups for a 12-week period under free-living conditions. The recruitment took place between August 2017 and May 2018 in Freising, Germany. The participants' eligibility was assessed with a detailed screening questionnaire. The detailed inclusion and exclusion criteria were described recently (18).

### Study Design and Intervention

The study was designed as a single-blinded (participant-blinded), randomized, controlled two-arm comparison. In total, 115 eligible participants were randomly assigned to either the treatment or placebo group in a 2:1 ratio. The intervention and control group included equal proportions of normal-weight individuals and of subjects with an elevated waist circumference (>102 cm in males and >88 cm in females) indicating an elevated cardiometabolic risk. Therefore, this group was also referred to as people with elevated “cardiometabolic risk.”

The primary outcome was to assess if recommendation and free provision of fiber-enriched products can increase fiber intake. Secondary outcomes included the acceptance of fiber-enriched products and changes of cardiometabolic risk factors, such as parameters of lipid and glucose metabolism as well as of microbiome composition.

**Figure 2:**
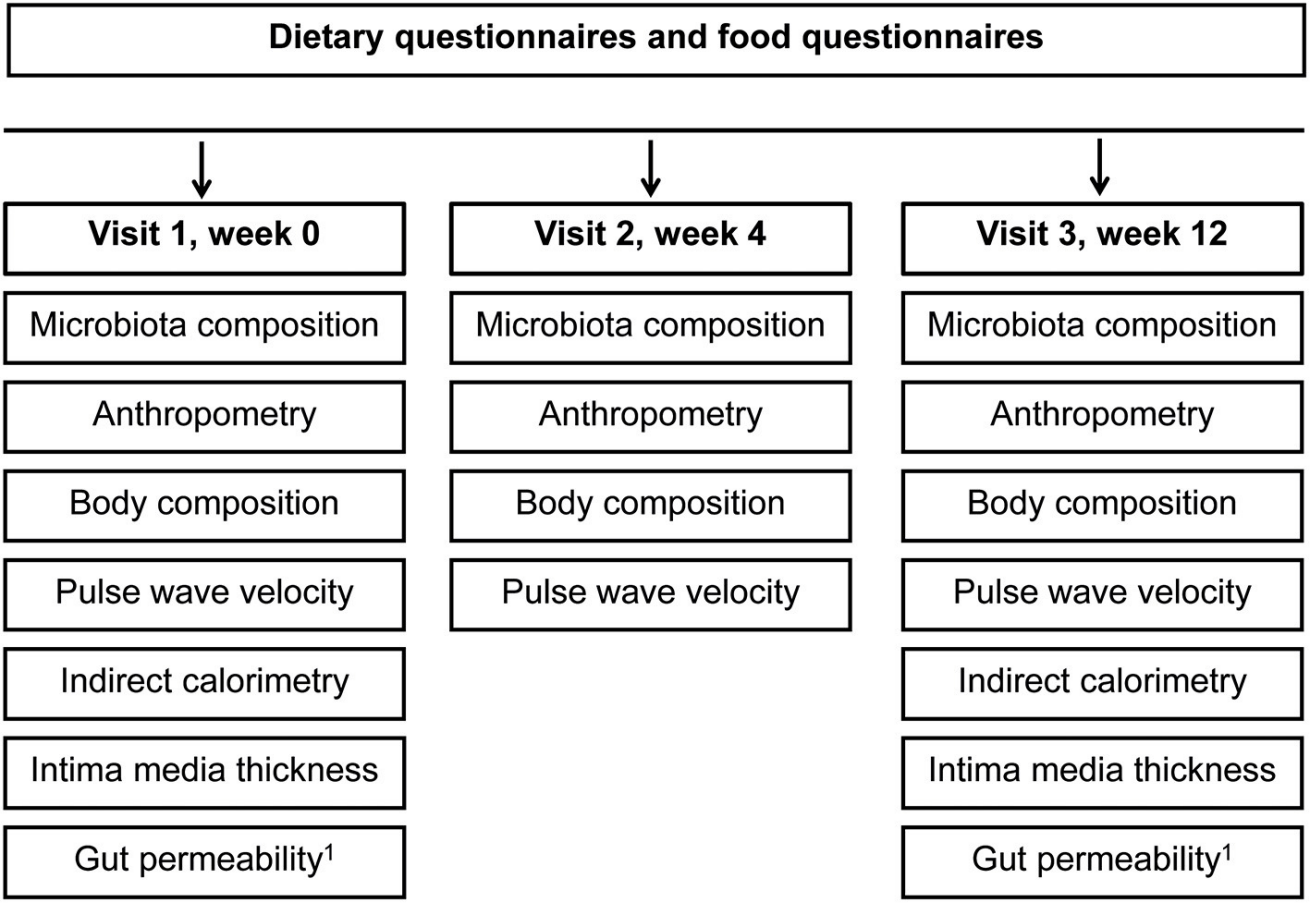
Timeline and examinations. ^1^Gut permeability was measured in a subgroup of participants (*n* = 35).

After recruitment, all participants were informed about the health value of fibers and encouraged to increase fiber intake. Participants allocated to the intervention group were invited to consume self-selected fiber-enriched foods for 12-weeks. The foods from the basket should provide 10 g of additional fiber per day. Participants allocated to the control group could select the identical food types without fiber enrichment from a portfolio of similar foods (Supplementary Table 1). Enrichment of food was mainly done with wheat fiber belonging to the insoluble dietary fiber and oat fiber which is a soluble fiber. The provided foods made up approximately one third of total caloric intake, and participants in both groups were instructed to stick to their usual diet. Participants were invited to come to the study center once a week to pick up the self-selected food items at defined amounts. All assessments and examinations during the three study visits (baseline, week 4, 12) are shown in Figure 2.

### Dietary Protocols

The study participants were instructed to record their food consumption three times for 1 week each (before, after 3 weeks, after 11 weeks of intervention) using a specific diary. The energy content and macronutrient composition of the diets were calculated using the OptiDiet Plus software (Version 5.1.2.046, GOE mbH, Linden, Germany). In addition, participants were asked to document the intake of the provided complimentary foods (with or without fiber enrichment) daily for the whole study period.

### Questionnaires

Participants received a questionnaire on the acceptance of the provided fiber-enriched and normal foods. The taste of the study products was rated with a 5-point numeric scale from 1 (“I don't like it at all”) to 5 (“I like it very much”). In addition, information on gastrointestinal symptoms was collected throughout the whole study. For this purpose, participants received a specific questionnaire at three time points: at baseline, after 4 weeks, and after 12 weeks. The questionnaire included six questions on GI symptoms: “Did you feel bloated during the past week?,” “Did you have flatulence problems during the past week?,” “Did you suffer from constipation during the past week?,” “Did you suffer from diarrhea during the past week?,” “Did you have a very loose stool during the past week?,” and “Did you have a very hard stool during the past week?” The rating was done using a 7-point numeric scale from 0 (not at all) to 6 (intense symptoms).

### Anthropometry and Body Composition

All anthropometric (height, weight, waist circumference) and clinical parameters were measured in the morning following an overnight fast using established standard operation procedures (SOPs). Body composition was measured using the Seca mBCA 515 device (Seca GmbH & Co KG, Hamburg, Germany).

### Cardiovascular Functions

Pulse wave analysis was performed using a specific analyzer (TensioMed, Budapest, Hungary). For quality assessment, measurements with a standard deviation ≥0.7 were repeated according to the instructions of the company. Moreover, intima-media thickness (IMT) was determined employing an ultrasound ACUSON X700 device (Siemens Healthcare GmbH, Erlangen, Germany) with a high-frequency VF16-5 probe. The measurement was performed with the subject in a supine position. IMT was determined during the peak-systole according to an ECG reading. Images were taken, centered about 10 mm below the carotid artery bulb.

### Blood Sampling

Blood samples were collected in the fasting state. Lipid parameters (total cholesterol, LDL cholesterol, HDL cholesterol, triglycerides), hsCRP, and insulin were analyzed in plasma by SynLab (Munich, Germany). Blood glucose concentrations were determined using a HemoCue Glucose 201+ device (plasma-calibrated, HITADO GmbH, Möhnesee, Germany). Insulin sensitivity was estimated according to the Homeostatic Model Assessment-Insulin Resistance (HOMA-IR) formula (19). Lipopolysaccharide binding protein and zonulin were assayed in plasma using commercially available ELISAs (LBP, R&D, Wiesbaden, Germany and Immundiagnostik AG, Bensheim, Germany, respectively).

### Gut Permeability

In addition to measurement of the gut permeability marker zonulin in plasma, gut barrier function was assessed in a subgroup of 35 participants from both groups by a sugar absorption test as described by Norman et al. (20). Participants received a sugar test solution following an overnight fast and after collecting a baseline urine sample. The 100 mL sugar test solution contained mannitol (5 g), lactulose (10 g), and sucrose (20 g), and 6 tablets of sucralose (333.3 mg/tablet). The subjects were instructed to collect their whole urine at time-defined intervals (0–5 h, 5–26 h). Urine was sampled in containers with sodium acid (0.002 g) as a preservative and stored at −20°C until analysis.

### Quantitation of Carbohydrates and Sugar Alcohols

Mannitol, lactulose, sucrose, and sucralose in urine samples were quantified through high-performance ion chromatography (HPIC), as described earlier with slight modifications (20). The internal standard solution (50 μL), containing turanose (Sigma Aldrich, Steinheim, Germany) and meso-erythritol (VWR, Darmstadt, Germany), was added to an aliquot of the urine sample (500 μL). After adding a solution of 5-sulfosalicylic acid (20% in water, 50 μL, Sigma Aldrich, Steinheim, Germany) and the mixed bed ion-exchange resin to remove proteins and salts, respectively, the aliquots were incubated, centrifuged, and diluted for further analysis. Subsequently, 10 μL of the diluted urine samples were injected on a 4 × 250 mm CarboPac PA1 anion-exchange column with 4 × 50 mm guard column of the same type (Thermo Fisher Scientific, Schwerte, Germany) at an oven temperature of 30°C and eluted using the mobile phases A (water), B (150 mmol/L sodium hydroxide) and C (150 mmol/L sodium hydroxide, 500 mmol/L sodium acetate) by applying following gradient at a flow rate of 1 mL/min: 0 min 14% B and 1% C, 13 min 14% B and 1% C, 40 min 100% B, 42 min 100% B, 43 min 14% B and 1% C, 60 min 14% B and 1% C. The compounds were detected by means of pulsed amperometric detection (PAD) on a conventional gold working electrode using a quadruple waveform. Carbohydrates were quantified via internal standards. Data acquisition and data evaluation were carried out using the Chromeleon Software 7.2 (Thermo Fisher Scientific, Schwerte, Germany).

### Microbiota Analysis

Three fecal samples were collected from each participant, at baseline and at the 4-week and 12-week visit. Samples were sequenced by targeting the V3V4 region of the 16S rRNA gene using the paired-end Illumina MiSeq sequencing technology. A detailed description of sample preparation and sequencing was published recently (21). Sequences with low read counts (<6,000 reads per sample) were resequenced. Demultiplexed FASTQ files were preprocessed using the IMNGS pipeline to generate operative taxonomic units (OTUs) with a 97% clustering using UPARSE v8.1.1861 (22) as well as with the DADA2 pipeline to generate ASVs with a 99% clustering (23). Taxonomic classification was performed by using the SILVA database (24). OTUs with a relative abundance <0.25% across all samples were removed to prevent the analysis of spurious OTUs (25). Amplicon sequence variants (ASVs) were considered for the final analysis of the 16S rRNA gene sequencing data.

### Statistical Analysis

According to the initial power calculation and based on 80 participants in the intervention and 40 in the control group, the study had a 99.7% power to detect a 10 g difference in fiber intake using a significance level of 0.05 and assuming a within-group standard deviation of 10 g. The assumed standard deviation was based on data from the Nationale Verzehrsstudie II and follow-up surveys (17). Considering the healthy participants and those with elevated waist circumference separately, the study had a power of 86% to detect a 10 g difference in each subgroup, using a Bonferroni correction for multiple comparisons.

Data were analyzed in the R programming environment. Results were presented as mean ± SD, and *p* < 0.05 were regarded as statistically significant. In the first step, mean differences were assessed between visits separately in both groups (control group and intervention group) by using a linear mixed model with random intercept based on the varying influence of the different study participants. For this *lmer* function from the package *lme4* was used. Tukey's Test was used as a *post-hoc* analysis to compare means of the different visits. Moreover, log-transformation were done to fit the assumption of normal distribution (triglycerideds, HDL-cholesterol, fasting insulin, fasting glucose, HOMA-IR, fiber). In the second step, control group and intervention group were compared at each time point (baseline, after 4 weeks, after 12 weeks of intervention) according to distribution by using *t*-test or Wilcoxon- signed ranked test. Finally, to assess the effect of time (different visits) and intervention (control group or intervention group) both factors were included in a linear mixed model with random intercept based on the varying influence of the different study participants. Regarding the analysis of microbiota composition, details were published recently (25).

### Functional Analysis

The function prediction based on 16S rRNA gene sequencing was performed by using PICRUSt2 (26). To determine differences between the groups, the R package ALEDx2 was used. Predicted abundances were centered-log transformed and significance was analyzed by using a generalized linear regression. Pairwise significance between visits, within one intervention group (Intervention or placebo), was assigned if *p* < 0.05 and effect size >0.2. Centered-log transformed values of the selected pathways were correlated with ASVs with a relative abundance >0.1 and a prevalence >10%. ASVs with a negative or positive correlation in at least one pairwise comparison > 0.5 and a significance *p* < 0.05 were selected to generating the heatmap.

## Results

### Baseline Characteristics of the Participants

One hundred and eight healthy individuals between the age of 40 and 65 years completed the study (Figure 1). Forty eight were males, and 60 were females. Seventy four subjects were randomized to the intervention group, 34 to the control group.

Baseline characteristics did not differ significantly between the intervention and control group and when comparing control group and intervention group on each visit (Table 1). All anthropometric parameters did not change during the 12-week study period in the intervention group. Considering only participants with cardiometabolic risk in the intervention group (Supplementary Table 2), participants showed a decrease in waist circumference (*p* < 0.01) and fat mass (*p* = 0.03). In contrast, in the control group, waist circumference (*p* < 0.01) and BMI (*p* = 0.04) increased slightly during 12 weeks (Supplementary Table 5).

**Table 1:** Baseline and follow-up characteristics of the participants and changes of anthropometric parameters during the study.

	**Control group**			**Intervention group**		
	**V1**	**V2**	**V3**	**V1**	**V2**	**V3**
***n***	34 (14 m, 20 f)			74 (34 m, 40 f)		
Age, years	52 ± 6			53 ± 7		
WC, cm	92.1 ± 15.0^a^	92.3 ± 14.6^a^	94.0 ± 13.9^b^	93.9 ± 12.9	93.7 ± 12.8	93.0 ± 12.2
Weight, kg	79.4 ± 16.4^a^	79.5 ± 16.7^a,b^	78.0 ± 17.0^b^	80.8 ± 16.3	80.7 ± 15.8	80.6 ± 15.6
BMI, kg/m^2^	26.4 ± 4.1^a^	26.5 ± 4.2^a,b^	26.6 ± 4.3^b^	27.1 ± 4.3	27.1 ± 4.2	27.1 ± 4.2
Fat mass, %	32.0 ± 6.5	32.0 ± 6.6	32.1 ± 6.7	32.5 ± 8.1	32.4 ± 8.0	32.0 ± 8.1
Fat free mass, %	68.0 ± 6.5	68.0 ± 6.6	67.9 ± 6.7	67.5 ± 8.1	67.6 ± 8.0	68.0 ± 8.1
Resting metabolic rate, kcal/day	1,569 ± 364	NA	1,656 ± 482	1,634 ± 419	NA	1,633 ± 350

*Data are presented as mean ± standard deviation. p < 0.05 were regarded as statistically significant. Mean differences were assessed between visits separately in both groups (control group and intervention group) by using a linear mixed model with random intercept based on the varying influence of the different study participants. Control group and intervention group were compared at each time point according to distribution by using t-test or Wilcoxon- signed ranked test. Labeled means in a row without a common superscript letter differ, p < 0.05. BMI, body mass index; V1, visit at baseline; V2, visit after four weeks of intervention; V3, visit after 12 weeks of intervention; WC, waist circumference*.

### Effect of the Intervention on Fiber Intake

The intervention group was instructed to include fiber-enriched foods of their choice in their normal diet (two items or portions per day), while the control group received a similar free selection of foods, but without fiber enrichment. Table 2 shows that the intervention group increased fiber intake significantly from 22.5 ± 8.0 to 34.0 ± 9.6 g/day after 4 weeks (*p* < 0.001) and to 36.0 ± 8.9 g/day after 12 weeks (*p* < 0.001), representing a fiber increase by 11 and 13 g/day, respectively. Compared to the control group, the intervention group increased their fiber intake significantly (*p* < 0.001).

**Table 2:** Dietary intake per day.

	**Control group**			**Intervention group**		
	**V1**	**V2**	**V3**	**V1**	**V2**	**V3**
***n***	34 (14 m, 20 f)			74 (34 m, 40 f)		
Energy intake, kcal/d	2,168 ± 672^a^	2,161 ± 556^a,b^	2,250 ± 598^b^	2,164 ± 511	2,106 ± 494	2,187 ± 435
Fat, %/d	36.0 ± 4.2	35.7 ± 3.8	35.0 ± 3.8	35.5 ± 5.2	35.4 ± 4.4	35.2 ± 5.5
Cholesterol, mg/d	310 ± 152^a^	256 ± 108^b^	265 ± 138^a,b^	332 ± 107^a^	254.8 ± 122^b^	272.6 ± 123^b^
Carbohydrates, %/d	42.3 ± 5.5^a^	44.5 ± 4.5^b^	45.0 ± 5.1^b^	42.1 ± 6.1	42.7 ± 5.0	42.8 ± 5.7^*^
Fiber, g/d	24.1 ± 8.7	22.6 ± 8.0	22.9 ± 6.8	22.5 ± 8.0^a^	34.0 ± 9.6^b^^*^	36.0 ± 8.9^b^^*^
Protein, %/d	15.3 ± 2.6^a^	14.7 ± 2.0^a,b^	14.0 ± 1.8^b^	16.6 ± 3.0^a^	15.5 ± 2.9^b^	15.1 ± 2.4^b^^*^
Alcohol, %/d	3.4 ± 4.5	2.5 ± 2.5	3.5 ± 4.2	3.1 ± 2.9	2.7 ± 2.9	3.1 ± 3.1

*Data are presented as mean ± standard deviation*.

*p < 0.05 were regarded as statistically significant. Mean differences were assessed between visits separately in both groups (control group and intervention group) by using a linear mixed model with random intercept based on the varying influence of the different study participants. Control group and intervention group were compared at each time point according to distribution by using t-test or Wilcoxon- signed ranked test. Labeled means in a row with a common superscript letter do not differ, p < 0.05*.

*V1, visit at baseline; V2, visit after four weeks of intervention; V3, visit after twelve weeks of intervention*.

* *Different from control, p < 0.05*.

**Figure 3:**
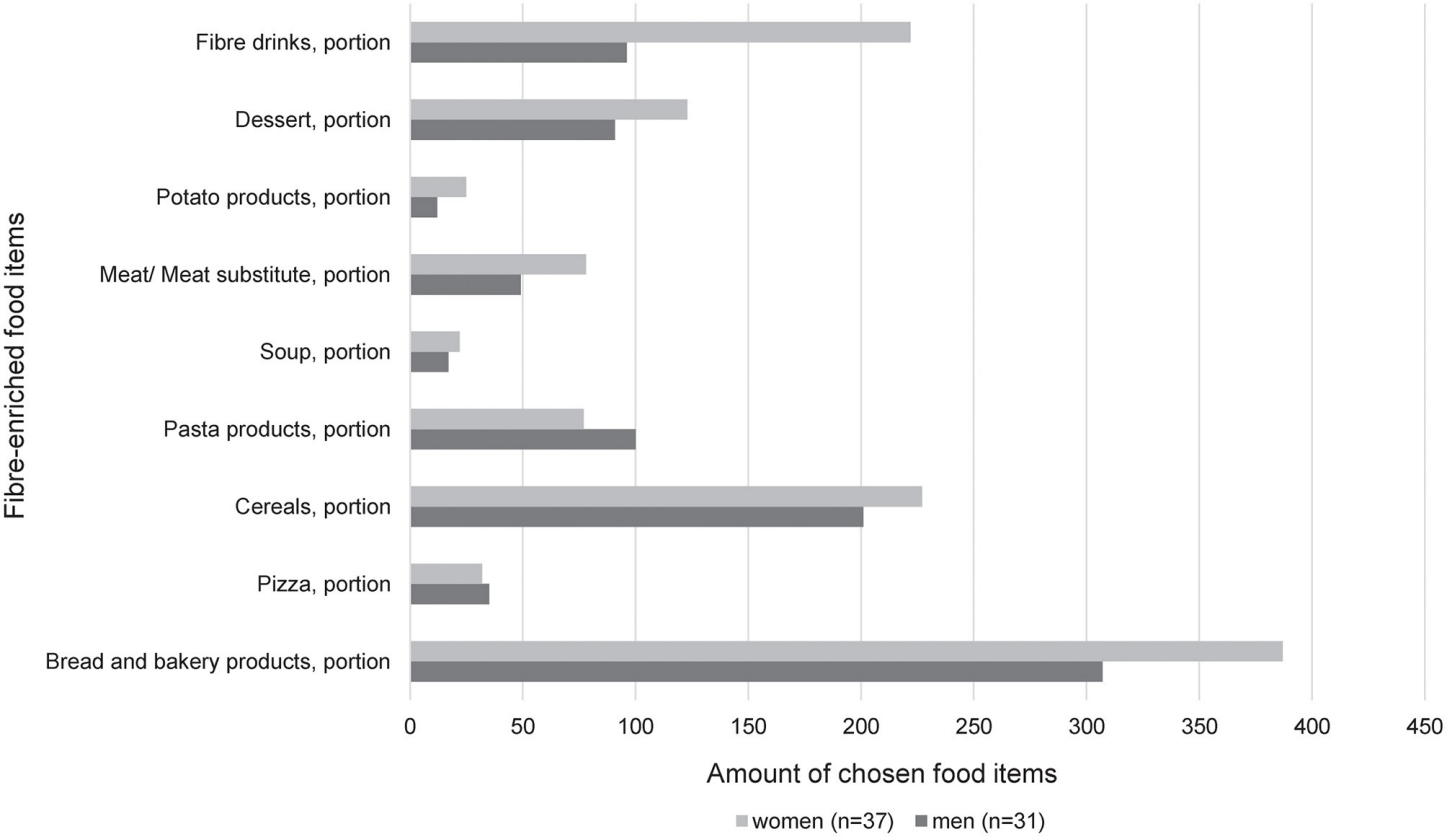
Relative contribution of the various fiber-enriched food groups by sex in the intervention group [women (*n* = 37); men (*n* = 31)]. The consumption of the different products was obtained 1 week before visit 2, respectively, visit 3. The portion size of each food item is listed in Supplementary Table 1.

The relative contribution of the various fiber-enriched food groups by sex is shown in Figure 3. Women reported a higher intake of fiber-enriched drinks compared to men (*p* = 0.02). Interestingly, the other products (dessert, potato products, meat/meat substitutes, soup, pasta products, cereals, pizza, and bread and bakery products) were almost equally consumed. Comparing the intervention and control group, pasta products (*p* = 0.032) and fiber drinks (*p* = 0.04) were significantly more frequently chosen in the intervention group.

Table 2 demonstrates the full dietary intake in both groups at baseline and after 4 and 12 weeks, respectively. Participants receiving fiber-enriched foods had no significant changes regarding total energy intake, fat, carbohydrate, and alcohol intake, whereas the intake of cholesterol (*p* < 0.001) and protein (*p* < 0.001) decreased within 12 weeks of intervention. As expected, the fiber intake of the participants in the control group remained unchanged during the 12 weeks; only the intake of energy (*p* = 0.04) and carbohydrates (*p* < 0.01) was modestly but significantly increased after the 12-week study period, whereas protein intake decreased (*p* < 0.01). Significant differences regarding the intake of carbohydrates (*p* = 0.01) and protein (*p* = 0.02) were found between the intervention and control group (Table 2). No distinction was made with regard to the physicochemical properties, since different fibers have overlapping properties.

### Impact of the Intervention on Cardiometabolic Risk Factors

The course of metabolic parameters from baseline to week 12 in both groups is presented in Table 3. Comparison between the three-time points in the intervention group revealed significant decreases in total cholesterol (*p* < 0.001), triglycerides (*p* = 0.03), LDL-cholesterol (*p* < 0.01), and fasting plasma insulin (*p* = 0.04). Slightly more pronounced effects were observed in the subgroup of subjects with increased waist circumference risk (Supplementary Tables 3, 4). In contrast, in the subgroup without cardiometabolic risk, only minor changes were seen (Supplementary Tables 6, 7). Compared to the control group, only heart rate was significantly lower in participants with cardiometabolic risk undergoing the intervention (all *p* < 0.05) (Supplementary Table 4). Overall, rather modest changes were found within and between groups. Intention-to-treat-analysis did not change the results.

**Table 3:** Changes of selected metabolic parameters in the intervention and control group during the 12-week study period.

	**Control group**			**Intervention group**		
	**V1**	**V2**	**V3**	**V1**	**V2**	**V3**
*N*	34 (14 m, 20 f)			74 (34 m, 40 f)		
Cholesterol, mg/dl	215 ± 43^a^	204 ± 27^b^	205 ± 34^b^	218 ± 32^a^	207 ± 32^b^	207 ± 31^b^
Triglycerides, mg/dl	101 ± 48	96.6 ± 48	102 ± 50	121 ± 70^a^	122 ± 108^a,b^	108 ± 53^b^
HDL-C, mg/dl	62.1 ± 20^a^	58.8 ± 17^b^	61.1 ± 19^a,b^	59.2 ± 16	57.8 ± 16	59.1 ± 16
LDL-C, mg/dl	132 ± 36	125 ± 27	128 ± 34	134 ± 30^a^	128 ± 31^b^	131 ± 30^a,b^
LDL/HDL	2.3 ± 0.8	2.3 ± 0.9	2.3 ± 0.8	2.5 ± 0.9	2.4 ± 0.9	2.4 ± 0.9
hsCRP, mg/dl	0.2 ± 0.2	0.2 ± 0.2	0.2 ± 0.2	0.2 ± 0.3	0.2 ± 0.2	0.2 ± 0.2
Fasting insulin, μU/ml	5.0 ± 3.7	5.4 ± 4.2	5.1 ± 3.8	6.3 ± 5.1^a^	6.4 ± 5.1^a^	5.5 ± 5.2^b^
Fasting glucose, mg/dl	95.6 ± 10.2	93.9 ± 7.0	95.0 ± 10.8	94.0 ± 8.5	95.0 ± 9.5	93.6 ± 9.6
HOMA-IR	1.2 ± 0.9	1.3 ± 0.9	1.2 ± 1.0	1.5 ± 1.0	1.5 ± 1.1	1.3 ± 1.3

*Data are presented as mean ± standard deviation. p < 0.05 were regarded as statistically significant. Mean differences were assessed between visits separately in both groups (control group and intervention group) by using a linear mixed model with random intercept based on the varying influence of the different study participants. Control group and intervention group were compared at each time point according to distribution by using t-test or Wilcoxon- signed ranked test. Labeled means in a row with a common superscript letter do not differ, p < 0.05*.

*hsCRP, high-sensitivity C-reactive protein; V1, visit at baseline; V2, visit after four weeks of intervention; V3, visit after 12 weeks of intervention*.

Table 4 shows the changes in cardiovascular functional parameter during the 12-week study period. There was a “borderline” significant difference at baseline between the two groups for systolic blood pressure and central systolic blood pressure. During the 12-week intervention period, there were no relevant changes in these parameters with the exception of central systolic blood pressure in the intervention group (from 124 ± 17 mmHg to 120 ± 14 mmHg, *p* = 0.02).

**Table 4:** Changes of cardiovascular functions in the intervention vs. control group.

	**Control group**			**Intervention group**		
	**V1**	**V2**	**V3**	**V1**	**V2**	**V3**
*n*	34 (14 m, 20 f)			74 (34 m, 40 f)		
**Blood pressure**					
Systolic blood pressure, mmHg	127 ± 15^a^	122 ± 15^b^	127 ± 18^a^	133 ± 14	131 ± 15^*^	130 ± 14
Diastolic blood pressure, mmHg	81 ± 9^a^	78 ± 11^b^	79 ± 11^a,b^	83 ± 8.0	82 ± 9.3^*^	82 ± 8.3
Heart rate, beats/minute	62 ± 9	63 ± 9	61 ± 10	61 ± 8.6	62 ± 9.7	60 ± 8.9
**Intima media thickness**					
Arteria right, mm	0.6 ± 0.1	NA	0.7 ± 0.2	0.7 ± 0.2	NA	0.7 ± 0.1
Arteria left, mm	0.6 ± 0.1	NA	0.7 ± 0.1	0.7 ± 0.2	NA	0.7 ± 0.2
**Pulse wave velocity**					
Augmentation index aortic, %	36.0 ± 13.2^a^	32.2 ± 12.6^b^	33.6 ± 12.7^a,b^	37.1 ± 13.1	36.9 ± 13.9	37.8 ± 13.6
Central systolic blood pressure, mmHg	115 ± 15	114 ± 15	114 ± 15	124 ± 17^a^	122 ± 16^a, b^,^*^	120 ± 14^b^,^*^
Pulse wave velocity, m/s	8.2 ± 1.4	8.4 ± 1.6	8.1 ± 1.6	8.7 ± 2.0	8.4 ± 1.6	8.7 ± 2.1

*Data are presented as mean ± standard deviation. p < 0.05 were regarded as statistically significant. Mean differences were assessed between visits separately in both groups (control group and intervention group) by using a linear mixed model with random intercept based on the varying influence of the different study participants. Control group and intervention group were compared at each time point according to distribution by using t-test or Wilcoxon- signed ranked test. Labeled means in a row with a common superscript letter do not differ, p < 0.05. V1, visit at baseline; V2, visit after four weeks of intervention; V3, visit after twelve weeks of intervention*.

* *Different from control, p < 0.05*.

### Effect of Increased Fiber Intake on the Microbiota

**Figure 4:**
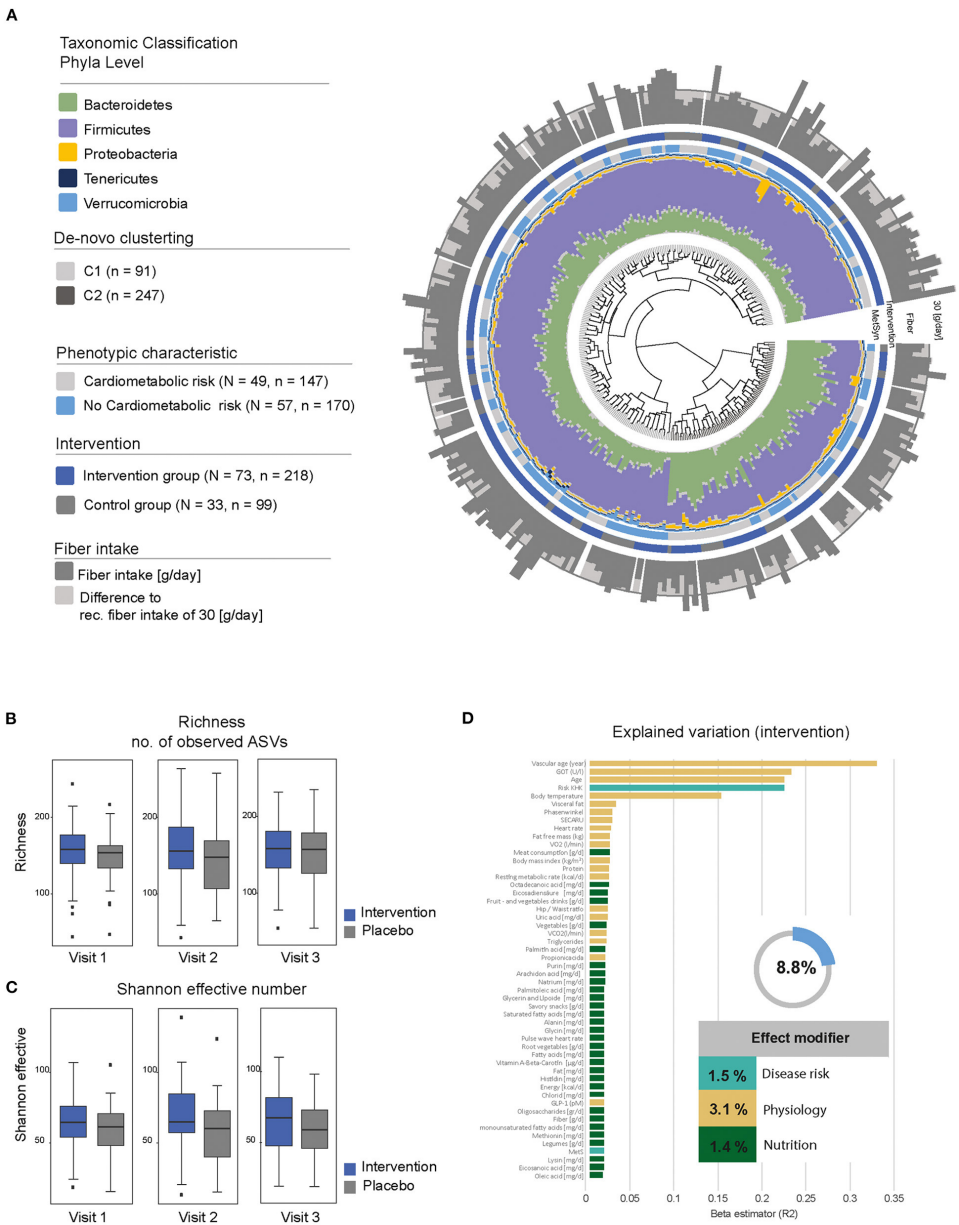
Description of the gut microbial composition. **(A)** Beta-diversity of the fecal microbiota in enable. The dendrogram shows similarities between microbiota profiles based on generalized UniFrac distances between subjects represented by individual branches. Unsupervised hierarchical clustering identified two main clusters of individuals (gray-scale next to branches). Individual taxonomic composition at the phylum level is shown as stacked bar plots around the dendrogram. The first ring indicates the presence of our criteria for “cardiometabolic risk” (gray, cardiometabolic risk; blue, no cardiometabolic risk); the second ring indicates the type of intervention (blue, fiber-enriched foods; gray, usual foods). Outer stacked barplot shows the fiber intake as well as the recommended threshold of 30 g/day (gray line). **(B,C)** Alpha-diversity stratified according to visit and intervention (blue, intervention; gray, control). Upper boxplots are showing richness; lower boxplots are showing bacterial diversity (Shannon effective number). **(D)** Explained variations in fecal microbiota composition by covariates. All variables shown had a significant influence (*P* ≤ 0.05) displayed as proportions of explained variations based on *R*^2^ calculated by multivariate analysis of Bray-Curtis dissimilarity. The top 52 variables are shown.

The microbial composition was dominated by the two main phyla Firmicutes and Bacteroidetes (Figure 4A). Unsupervised clustering resulted in three distinct groups, which showed a significant difference in the relative abundance of *Bacteroides* (C2), *Prevotella* and *Ruminocccus* (C1), which are known to be associated with diet (27). Individuals with the C2 cluster showed a reduced amount of fiber intake (mean 25.8 ± 8.79 g/day) compared to C1 (mean 28.7 ± 10.77 g/day) and C3 (mean 28.9 ± 9.82 g/day). The proportion of individuals at cardiometabolic risk was higher in C2, which resulted again in a reduced *alpha*-diversity. Overall differences in the bacterial composition of the gut can be explained by variables related to physiology (3.1%), disease risk (1.5%), and nutrition (1.4%) and resulted in an overall effect modifier of 8.8% (Figure 4D).

**Figure 5:**
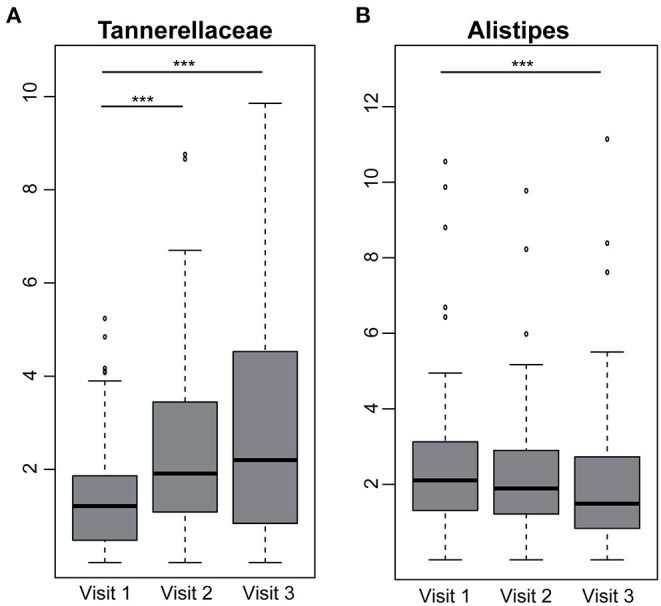
Differences between visits in individuals receiving fiber-enriched foods. Boxplots are showing the relative abundance values of the family **(A)** *Tannerellaceae* and the genus **(B)** *Alistipes*. Significance is shown between groups (Benjamin Hochberg adj. *p* < 0.05), ***p < 0.001.

The taxonomic classification based on phyla level between individuals with and without fiber intervention showed a heterogeneous distribution with no significant differences. Nevertheless, there was an increased relative abundance in *Bacteroidetes* in individuals with elevated waist circumference (35.8 vs. 30.2%) and a decreased abundance in *Firmicutes* (58.4 vs. 63.0%). These differences were also seen in the number of observed amplicon sequence variants (ASVs) with a decreased richness (302 elevated waist vs. 331 normal; *p* = 0.0001) as well as Shannon effective counts (74 elevated waist vs. 88 normal; *p* = 8.022e-07) (Figures 4B,C). An increased fiber consumption resulted in an increased relative abundance of *Tannerellaceae* observed between visit 1 and visit 2 as well as between visit 1 and visit 3 (Figure 5). After the third visit, a reduced relative abundance in *Alistipes* was seen, whereas an increased abundance in *Alistipes* was associated with inflammatory diseases and non-alcoholic fatty liver disease (NAFLD) (28). Additionally, some taxa also showed differences between visits, when individuals who received fiber-enriched foods were compared with individuals who received usual foods (Supplementary Figure 1).

### Metabolic Pathways Associated Increased Fiber Intake

**Figure 6:**
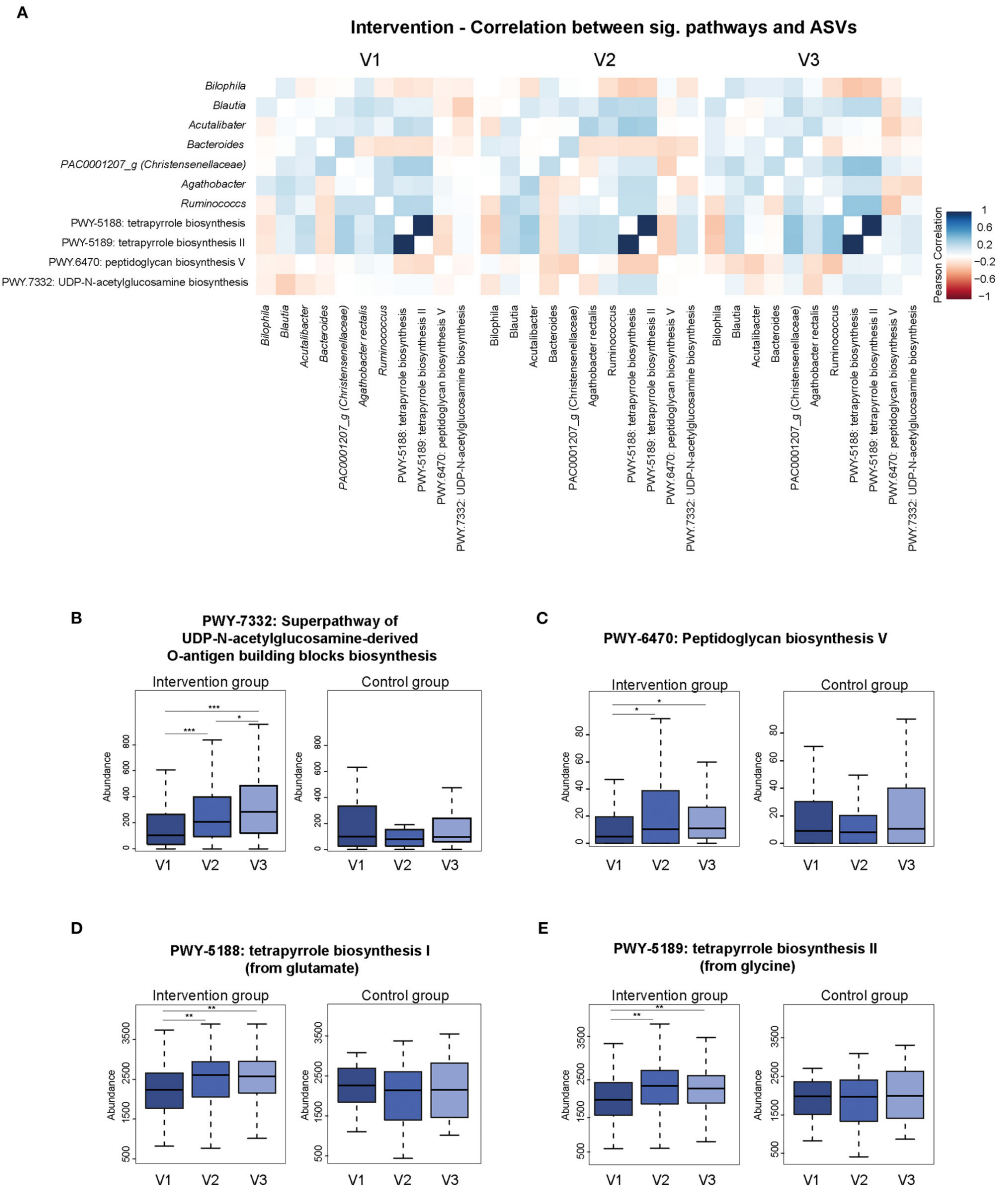
The Association of predicted functional pathways and ASVs. **(A)** Predicted functional pathways (PiCRUST2) which are significantly different between visits in the intervention group. Heatmaps show significant correlation between selected pathways (Pearson's *R* ≥ 0.3; Pearsons's *R* ≤ −0.3) and ASVs. Data was stratified according to visits. Strong negative correlations are shown in red and positive correlation are shown in blue. **(B–E)** Panels show significant differences in abundances of predicted pathways between visits within the intervention group compared to the placebo group. *p < 0.05, **p < 0.01, ***p < 0.001.

Predicted functional pathways derived by 16S rRNA gene sequences showed that different pathways seemed to vary between visits comparing participants who underwent the intervention and those who did not get a fiber enriched diet. Overall, 13 pathways were significantly different between visits in the intervention group. Out of these, 9 pathways were also found to be different in the placebo group. The remaining four pathways significantly correlated with 7 ASVs (Figure 6A). PWY7332—a biosynthesis pathway associated with N-acetylglucosamine—also showed a steady significant increase in abundance over time (Figure 6B). Its correlation with *Blautia* changed from a negative to a positive correlation. The increased abundance of Acetylglucosamine biosynthesis was accompanied by the appearance of a negative correlation with *Agathobacter*. Peptidoglycan biosynthesis (PWY.6470) correlated positively with the genera *Agathobacter* and *Ruminococcus*, but changed over time to a negative association (Figure 6C). Overall, the abundance of this pathway increased over time, starting at a very low baseline abundance but increased after the intervention had started. The two tetrapyrrole biosynthesis pathways (PWY-5188, PWY-5189) increased in their abundance between the first and second visit and correlated negatively with the two genera *Bilophila* and *Bacteroides* (Figures 6D,E). Additionally, predicted functional pathways which were significantly different between visits in the placebo group were depicted in Supplementary Figure 2.

### Fiber Intake and Gut Permeability

In a subgroup of 35 participants, gut permeability was measured before and after 12 weeks of intervention. Supplementary Table 8 summarizes the results obtained using different approaches for the measurement of gut permeability. The paracellular gut permeability markers, lipopolysaccharide-binding protein (LBP), zonulin and lactulose, was comparable at baseline and did not change after 12 weeks in both groups. Furthermore, the percentage urine recovery of sucrose, mannitol, and sucralose remained unchanged before and after treatment.

### Rating of the Fiber-Enriched Products

Overall, most participants rated the taste of the study products as enjoyable. In both groups, the most liked foods included pretzel breadstick, pizza salami, and pizza vegetarian (Table 5). Participants in the control group rated bread and bread roll better than participants in the intervention group, and the fiber-enriched bread roll was the least favorite product in the portfolio for the intervention group. The least favorite food item in both groups was meatloaf, as nearly half of the participants either disliked it or answered to “neither like nor dislike it” (Table 5).

**Table 5:** Rating of selected study products by participants of the intervention group **(A)** and participants of the control group **(B)**.

**Intervention group (A)**	**Bread roll**	**Bread**	**Pretzel breadstick**	**Pizza salami**	**Pizza vegetarian**	**Meat loaf**
	***n* = (54)**	***n* = (63)**	***n* = (62)**	***n* = (52)**	***n* = (54)**	***n* = (37)**
	***n* (%)**	***n* (%)**	***n* (%)**	***n* (%)**	***n* (%)**	***n* (%)**
I like it	25 (46.3)	39 (61.9)	43 (69.4)	36 (69.2)	40 (74.1)	21 (56.8)
Neither like nor dislike	16 (29.6)	12 (19.0)	12 (19.4)	7 (13.5)	9 (16.7)	7 (18.9)
I don't like it	13 (24.1)	12 (19.0)	7 (11.3)	9 (17.3)	5 (9.3)	9 (24.3)
**Control group (B)**	**Bread roll**	**Bread**	**Pretzel breadstick**	**Pizza salami**	**Pizza vegetarian**	**Meat loaf**
	***n*** **=** **(26)**	***n*** **=** **(33)**	***n*** **=** **(30)**	***n*** **=** **(23)**	***n*** **=** **(25)**	***n*** **=** **(20)**
	***n*** **(%)**	***n*** **(%)**	***n*** **(%)**	***n*** **(%)**	***n*** **(%)**	***n*** **(%)**
I like it	19 (73.1)	29 (87.9)	23 (76.7)	15 (65.2)	21 (84.0)	11 (55.0)
Neither like nor dislike	2 (7.7)	1 (3.0)	6 (20.0)	8 (34.8)	4 (16.0)	3 (15.0)
I don't like it	5 (19.2)	3 (9.1)	1 (3.3)	0 (0.0)	0 (0.0)	6 (30.0)

### Gastrointestinal Tolerance of Fiber-Enriched Products

At baseline, the most frequently reported gastrointestinal symptoms were flatulence (intervention group 56.8 vs. 52.9% in the control group) and bloating (50.0 vs. 55.9%, respectively). Symptoms were mostly described as mild to moderate. At week 12, the overall percentage of participants reporting flatulence increased to 71.6% in the intervention group, including (*n* = 5) participants who reported strong symptoms. In the control group, the overall percentage of flatulence symptoms was 58.8% at 12 weeks, and no strong symptoms were reported. Regarding bloating, 55.4% of participants in the intervention group reported some symptoms at 12 weeks, including 4 participants, who reported intense symptoms. In the control group, the percentage of participants who suffered from bloating at week 12 decreased to 44.1%. Concerning other gastrointestinal complaints such as loose stool, diarrhea and constipation no differences and changes from baseline to week 12 in both groups were seen (data not shown).

## Discussion

The results of this intervention study clearly indicate that offering fiber-enriched popular foods leads to an increase of fiber intake by more than 10 g/day compared to a control group receiving similar products without fiber enrichment. Participants were asked to stick to their usual dietary habits and no further recommendations for a healthy diet were given. It is necessary to mention that we did not separate between certain physico-chemical properties. Volunteers were free to choose their foods without paying attention whether the fiber type was more soluble/insoluble or viscous/non-viscous. Many fiber types have overlapping physicochemical properties. Therefore, it is not possible to assign observed effects to distinct fiber types.

A unique feature of this study was that fiber-enriched foods were offered that are already available in food markets or were specifically developed in a research project to increase the health value of popular convenience foods by adding fiber (18, 29). Thereby, a broad portfolio of fiber-enriched food items was composed that reflects common dietary habits of the German population and is not necessarily consistent with official dietary guidelines. Here, this alternative strategy was shown to improve an important component of diet quality, as previous efforts to increase fiber intake by promoting a higher intake of vegetables, fruits or whole-grain bread have turned out to be not very effective (30).

It is interesting to note that the majority of participants in the intervention group reported that they enjoyed the provided food items. It was a specific goal of this study to offer fiber-enriched foods with high sensory qualities and, at best, they should be undistinguishable from standard foods, as recently shown in a reformulation study from our group (29).

However, it is well-known that the palatability of food items may be influenced by the kind of food, the amount of added fiber and its physiochemical properties, the food texture and its organoleptic properties as demonstrated in previous studies (31–34). In our study, the participants could select from a variety of fiber-enriched products. The analysis of the product ratings revealed that the standard products were rated only slightly better than the fiber-enriched alternatives. This small difference may not be really relevant, as there was no difference in dropout rates in both groups during the 12-week study period suggesting a high acceptability of the fiber-enriched products. However, participants of the intervention group reported a moderate increase in gastrointestinal symptoms due to the higher fiber intake without affecting adherence.

Moreover, we assessed the impact of fiber-enriched foods on body weight, lipids, insulin sensitivity, and cardiovascular functions. In the current study, the increase of fiber intake did not promote a change in body weight. Previous studies established a weak inverse relationship between dietary fiber intake and changes in body weight (35, 36). For instance, in the prospective EPIC cohort study, an increase in dietary fiber intake by 10 g/day was associated with an annual weight change of −39 g/year (95% CI: −71, −7 g/year) (36). Therefore, it is obvious that the impact of a high fiber intake on body weight is generally rather modest. Another explanation could be that the intervention period was too short to produce effects on body weight.

Furthermore, the increased fiber intake did not confer short-term metabolic benefits, as there were no relevant changes in total cholesterol, LDL-cholesterol, and triglycerides as well as HOMA-IR as a marker of insulin sensitivity. These findings are in line with results from several previous studies (1, 37). In addition, the increase of fiber intake resulted in a significant decrease of fasting insulin, whereas HOMA-IR did not change significantly after 12 weeks of intervention. A previous study suggested that the effect of insoluble fiber on insulin resistance may depend on the ingested amount (38). In the latter study, 31.2 g/day of insoluble fiber was given to women with overweight or obesity and normal glucose tolerance which resulted in an improved insulin sensitivity compared to the additional 10–12 gram of fiber in the present study.

The measurement of cardiovascular functional parameters revealed a modest decrease in central systolic blood pressure. This was not unexpected, as a recent study reported that some fiber products may have a short-term antihypertensive effect on blood pressure (39). It is known that dietary fibers may interact with cells of the immune barrier in the small intestine (40), strengthen the mucus layer and enhance the barrier function of epithelial cells (41). In the current study, we were interested to investigate the impact of increased dietary fiber intake on gut permeability in a subgroup of participants. However, there was no measurable effect of the increased dietary fiber intake on gut permeability. There are only a few studies on this aspect with some positive effects of dietary fiber on gut permeability, however, mainly in non-healthy individuals (42, 43). More studies in this field are needed also considering the type and the amount of fiber intake.

In contrast, more data is available on the effects of dietary fibers on the growth and function of intestinal microbiota communities (44). Therefore, we were interested to investigate, if our approach to increase fiber intake has an effect on microbiota composition. The results of this analysis indicate that individuals with additional fiber intake showed an increase of members of the family *Tannerellaceae*. This result confirms the previously reported association of *Tannerellaceae* with obesity (45) and hypertension (46) and should be considered in the analysis of diet-related metabolic health. Members of the family *Tannerellaceae* are known to be involved in succinate production, which is associated with intestinal inflammation (47) and metabolic health (48, 49). However, it is important to be aware that only a minor proportion of the microbial diversity can be explained by environmental factors including diet (25, 50).

The prediction of functional pathways allowed us to analyse functional pathways derived by 16S rRNA gene sequences. The results of this showed that the increase of the two tetrapyrrole biosynthesis pathways (PWY-5188, PWY-5189) reduces the presence of *Bilophila* and *Bacteroides*. The two genera were associated with high fat diet and metabolic dysfunction and weight loss under lifestyle intervention (51, 52). Moreover, the Peptidoglycan biosynthesis (PWY.6470) were linked with the genera *Agathobacter* and *Ruminococcus* which increased after the intervention has started. *Agathobacter* include butyrate-producing species and *Ruminococcus* include degraders of complex dietary and host-derived polysaccharides (53). Overall, the association between functional pathways and 16S rRNA gene sequences reflected the reduction of high fat food and increase of fiber-enriched food.

The strength of our study is the pragmatic approach in a real world setting. It is obvious from our experience that fiber enrichment of popular convenience foods may be a simple way to increase fiber intake in the general population, provided a similar taste and appealing appearance. Another strength of this study may be that a variety of methods were applied to get a broad picture including the perspective of the consumers. Such human studies may also have limitations such as the assessment of dietary intake that was based on self-reporting of the participants. Although the provision of fiber-enriched and standard foods was documented, it cannot be excluded that the consumption of products was shared with family members. The rating of the fiber-enriched food products may have been influenced by the free provision to the participants, but the situation was the same in the control group. Although the recruitment used channels addressed to the general population, it is also possible that particularly health-conscious individuals participated. Therefore, the findings may not be generalizable.

In conclusion, the results of this study strongly suggest that fiber enrichment of popular foods including convenience foods may be a simple and effective method to increase fiber intake to the recommended level. Along this line, a modest improvement of some cardiometabolic risk factors can be expected in a rather healthy group of volunteers. Therefore, improving the health quality of popular convenience foods, could become a novel and effective strategy to improve the overall quality of the habitual diet in the population, especially in people with cardiometabolic diseases. Additional studies are needed to further explore this approach.

## Data Availability Statement

The datasets generated for this study are available on request to the corresponding author. The accession number for the raw sequencing data in this paper are available via Sequence Read Archive (SRA: PRJNA701859). Software used to analyze the data are either freely or commercially available. Source code data are available from the corresponding author on request.

## Ethics Statement

The studies involving human participants were reviewed and approved by Ethical Committee of the Faculty of Medicine of the Technical University of Munich. The patients/participants provided their written informed consent to participate in this study.

## Author Contributions

HH and TS designed research. RR conducted research. TH provided essential reagents. SD and BB analyzed gut permeability marker. SR and DH analyzed microbiota data. BB performed statistical analysis. KP analyzed data from questionnaires. HH, TS, and BB had primary responsibility for final content. All authors read and approved the final version of the manuscript.

## Funding

This work was funded by a grant from the German Ministry for Education and Research (BMBF, Grant No. 01EA1409A). There was no influence on the design, conduct, data analysis, and interpretation from the funding agency and the industrial partners. The preparation of this paper was supported by the enable Cluster and is cataloged by the enable Steering Committee as enable 89 (http://enable-cluster.de).

## Conflict of Interest

The authors declare that the research was conducted in the absence of any commercial or financial relationships that could be construed as a potential conflict of interest.

## Publisher's Note

All claims expressed in this article are solely those of the authors and do not necessarily represent those of their affiliated organizations, or those of the publisher, the editors and the reviewers. Any product that may be evaluated in this article, or claim that may be made by its manufacturer, is not guaranteed or endorsed by the publisher.

## Abbreviations

ASVs: Amplicon sequence variant
DGE: German Nutrition Society
HOMA-IR: homeostatic model assessment-Insulin Resistance
HPIC: high-performance ion chromatography
hsCRP: high-sensitivity C-reactive protein
IMT: intima-media thickness
LBP: lipopolysaccharide binding protein
LDL: low-density lipoprotein cholesterol
NAFDL: non-alcoholic fatty liver disease
OTU: operative taxonomic unit
PAD: Pulsed amperometric detection
RCTs: randomized controlled intervention studies
rRNA: ribosomal ribonucleic acid
SOP: standard operation procedure
WC: Waist circumference.
