# Supplementary material for: Offering Fiber-Enriched Foods Increases Fiber Intake in Adults With or Without Cardiometabolic Risk: A Randomized Controlled Trial
Source: Front Nutr. 2022 Feb 16;9:816299. doi: 10.3389/fnut.2022.816299 (PMC8890034; doi:10.3389/fnut.2022.816299)
Supplement: Supplementary file 1 [file Data_Sheet_1.pdf]

Supplementary Tables

Table 1. List of fiber-enriched food items provided by food industry including types of fiber

Fiber- enriched food items	Types of fibre	Portion size of food item	Amount of fiber/portion
**Bread and bakery products**			
Bread	wheat fiber, whole grain spelt flour	100 g	10.7 g
Bread roll	wheat fiber	64 g	4.1 g
Pretzel breadstick	wheat fiber	71 g	4.2 g
Chocolate cake	peas fiber, inulin	100 g	6.0 g
Muffin	wheat fiber, polydextrose	97 g	2.2 g
**Cereals**			
crunchy	whole grain oats, wheat germs, wheat whole grain	60 g	7.8 g
with dried strawberries	whole grain oats, wheat germs, wheat whole grain	60 g	7.2 g
Cornflakes	whole grain, whole grain oats	40 g	1.7 g
Clusters Almond	whole grain wheat flour	60 g	5.2 g
**Soup**			
Pea soup	oat fiber	375 ml	5.1 g
Asparagus cream soup	oat fiber	500 ml	6.0 g
Mushroom cream soup	oat fiber	500 ml	6.0 g
**Pasta products**			
Lasagne	oat fiber	633 g	6.0 g
Bolognese	oat fiber	431 g	5.4 g
Instant soup	oat fiber	243 g	5.4 g
Napoli sauce	oat fiber	154 g	4.0 g
Carbonara Sauce	oat fiber	194 g	2.1 g
**Potato products**			
Mashed potatoes	oat fiber	300 g	7.7 g
Instant soup	oat fiber	250 g	5.5 g
**Pizza**			
Pizza salami	wheat fiber, powder cellulose, inulin	320 g	20.5 g
Pizza vegetarian	powder cellulose, inulin	385 g	23.5 g

**Meat/ Meat substitute**			
Meat loaf	wheat fiber, resistant dextrin	75 g	4.7 g
Vegetarian fillet stripes	peas fiber, cellulose	75 g	6.8 g
Vegetarian cutlets	citrus fiber	90 g	3.9 g
**Dessert**			
Fruits	resistant dextrin, barley flakes, galacto-oligosaccharide, wheat aleuron	150 g	10.2 g
Chocolate mousse	resistant starch, inulin	85 g	5.4 g
Fibre drink (chocolate taste)	oat fiber, dextrin, psyllium husk fiber	200 ml	10.9 g
Fibre drink (vanilla taste)	oat fiber, dextrin, psyllium husk fiber	200 ml	10.5 g
Fibre drink (orange taste)	oat fiber, dextrin, psyllium husk fiber	200 ml	10.5 g

Fibers were approved by the U.S. Food and Drug Administration and achieved GRAS-status (Generally recognized as Safe).

Table 2. Baseline and follow-up characteristics of participants with cardiometabolic risk

	Control group			Intervention group		
	V1	V2	V3	V1	V2	V3
n		13 (6m, 7f)			37 (17m, 20f)	
Age, years		52±7			54±7	
WC, cm	105.3±9.1	104.9±9.6	105.9±8.2	103.6±7.9^{a}	102.9±7.8^{a}	101.1±8.8^{b}
Weight, kg	92.4±12.0	92.6±12.3	93.3±13.0	90.3±14.2^{a}	90.1±13.2^{a,b}	89.9±12.8^{b}
BMI, kg/m²	30.5±2.0	30.6±2.1	30.9±2.4	30.4±2.7^{a}	30.4±2.5^{a,b}	30.3±2.6^{b}
Fat mass, %	37.0±5.2	36.9±5.3	37.2±5.2	37.5±6.3^{a}	37.2±6.5^{a,b}	36.7±7.1^{b}
Fat free mass, %	63.0±5.2	63.1±5.3	62.8±5.2	62.5±6.3^{a}	62.8±6.5^{a,b}	63.3±7.1^{b}
Resting metabolic rate, kcal/day	1812±304	NA	1901±3729	1758±438	NA	1725±378

Data are presented as mean ± standard deviation. *p*-values < 0.05 were regarded as statistically significant. Mean differences were assessed between visits separately in both groups (control group and intervention group) by using a linear mixed model with random intercept based on the varying influence of the different study participants. Control group and intervention group were compared at each time point according to distribution by using t-test or Wilcoxon- signed ranked test. Labeled means in a row with a common superscript letter do not differ, $p < 0.05$. BMI, body mass index; WC, waist circumference; V1, visit at baseline; V2, visit after four weeks of intervention; V3, visit after twelve weeks of intervention; WC, waist circumference.

Table 3. Metabolic characteristics of participants with cardiometabolic risk

	Control group			Intervention group		
	V1	V2	V3	V1	V2	V3
n		13 (6m, 7f)			37 (17m, 20f)	
Cholesterol, mg/dl	209±34.9	200±30.6	202±36.6	218±30.6[a]	206±32.5[b]	206±32.1[b]
Triglycerides, mg/dl	125±64.1	126±58.1	126±66.5	140±78.7[a]	124±75.9[b]	122±60.1[b]
HDL-c, mg/dl	55.2±10.2[a]	51.8±7.0[b]	54±8.9[a,b]	56.9±12.7	54.6±16.2	55.9±16.4
LDL-c, mg/dl	133±37.1	126±30.3	132±34.6	137±29.1	131±28.9	134±31.3
LDL/HDL	2.5±0.8	2.5±0.8	2.5±0.7	2.7±1.0	2.6±1.0	2.6±1.0
hsCRP, mg/dl	0.3±0.2	0.3±0.2	0.3±0.2	0.3±0.3	0.3±0.2	0.3±0.3
Fasting insulin, µU/ml	7.1±4.3	8.2±4.8	8.1±4.3	7.9±4.7[a]	8.2±4.4[a]	6.7±4.5[b]
Fasting glucose, mg/dl	99.7±9.2[a]	92.7±7.5[b]	97.9±13.2[a,b]	96.6±9.2	98.2±9.2	95.2±11.1
HOMA-IR	1.7±1.1	1.9±1.1	2.0±1.3	1.9±1.1[a]	2.0±1.1[a]	1.6±1.3[b]

Data are presented as mean ± standard deviation. *p*-values < 0.05 were regarded as statistically significant. Mean differences were assessed between visits separately in both groups (control group and intervention group) by using a linear mixed model with random intercept based on the varying influence of the different study participants. Control group and intervention group were compared at each time point according to distribution by using t-test or Wilcoxon- signed ranked test.Labeled means in a row with a common superscript letter do not differ, $p < 0.05$. hsCRP, high-sensitivity C-reactive protein; V1, visit at baseline; V2, visit after four weeks of intervention; V3, visit after twelve weeks of intervention.

Table 4. Cardiovascular function of participants with cardiometabolic risk

	Control group			Intervention group		
	V1	V2	V3	V1	V2	V3
n	13 (6m, 7f)			37 (17m, 20f)		
**Blood pressure**						
Systolic blood pressure, mmHg	136±13.8	133±11.2	134±14.5	138±15	135±13.7	134±12.0
Diastolic blood pressure, mmHg	87±9.1	84±10.2	85±9.3	84±7.7	83±8.5	83±8.1
Heart rate, beats/minute	68±6.4	69±7.0	69±8.6	63±9.1*	64±8.6*	61±9.2*
**Intima media thickness**						
Arteria right, mm	0.7±0.1	NA	0.7±0.2	0.7±0.2	NA	0.7±0.1
Arteria left, mm	0.7±0.1	NA	0.7±0.1	0.8±0.2	NA	0.7±0.1
**Pulse wave velocity**						
Augmentation index aortic, %	34.0±11.4[a]	26.9±10.9[b]	28.3±10.9[b]	37.6±12.3	36.6±13.3*	36.8±13.9
Central systolic blood pressure, mmHg	122±9.5	118±15.0	117±14.2	128±17[a]	126±14.6[a,b]	123±12.1[b]
Pulse wave velocity, m/s	8.8±1.6	8.8±1.9	8.7±2.1	9.0±2.0	8.5±1.3	8.9±2.0

Data are presented as mean ± standard deviation. *p*-values < 0.05 were regarded as statistically significant. Mean differences were assessed between visits separately in both groups (control group and intervention group) by using a linear mixed model with random intercept based on the varying influence of the different study participants. Control group and intervention group were compared at each time point according to distribution by using t-test or Wilcoxon- signed ranked test. Labeled means in a row with a common superscript letter do not differ, $p < 0.05$. V1, visit at baseline; V2, visit after four weeks of intervention; V3, visit after twelve weeks of intervention. *, different from control, p < 0.05.

Table 5. Baseline and follow-up characteristics of participants without cardiometabolic risk

	Control group			Intervention group		
	V1	V2	V3	V1	V2	V3
n		21 (8m, 13f)			37 (17m, 20f)	
Age, years		52±6			53±6	
WC, cm	83.9±11.6[a]	84.5±11.3[b]	86.6±11.3[b]	84.2±9.0	84.4±9.6	84.9±9.4
Weight, kg	71.3±13.3	71.5±13.9	71.7±13.8	71.2±12.1	71.3±12.1	71.3±12.2
BMI, kg/m²	23.9±2.8	23.9±2.9	24.0±2.9	23.9±2.6	23.8±2.6	23.9±2.6
Fat mass, %	29.2±5.5	28.9±5.4	28.9±5.4	27.4±6.4	27.6±6.3	27.6±6.4
Fat free mass, %	70.8±5.5	71.1±5.4	71.1±5.4	75.6±6.4	72.4±6.3	72.4±6.4
Resting metabolic rate, kcal/day	1423±321	NA	1496±505	1521±372	NA	1542±298

Data are presented as mean ± standard deviation. *p*-values < 0.05 were regarded as statistically significant. Mean differences were assessed between visits separately in both groups (control group and intervention group) by using a linear mixed model with random intercept based on the varying influence of the different study participants. Control group and intervention group were compared at each time point according to distribution by using t-test or Wilcoxon- signed ranked test. Labeled means in a row without a common superscript letter differ, $p < 0.05$. BMI, body mass index; V1, visit at baseline; V2, visit after four weeks of intervention; V3, visit after twelve weeks of intervention; WC, waist circumference.

Table 6. Metabolic characteristics of participants without cardiometabolic risk

	Control group			Intervention group		
	V1	V2	V3	V1	V2	V3
n		21 (8m, 13f)			37 (17m, 20f)	
Cholesterol, mg/dl	218±47.9	206±25.7	207±33.8	217±32.9[a]	208±32.5[b]	207±30.2[b]
Triglycerides, mg/dl	85.4±27.1	78.4±28.1	86.0±27.9	106±54.0	120±131	93.6±41.1
HDL-c, mg/dl	66.4±23.3	63.2±20.4	65.5±22.8	61.6±14.5	60.6±14.9	62.2±14.2
LDL-c, mg/dl	131±36.1	125±26.0	125±33.4	131±31.2	124±32.2	128±29.3
LDL/HDL	2.2±0.8	2.2±0.9	2.1±0.8	2.3±0.78	2.2±0.8	2.2±0.8
hsCRP, mg/dl	0.1±0.1	0.1±0.1	0.1±0.1	0.2±0.3	0.1±0.1	0.1±0.2
Fasting insulin, µU/ml	3.8±2.7	3.7±2.6	3.2±1.8	4.6±5.0	4.8±5.2	4.3±5.6
Fasting glucose, mg/dl	93.1±10.2	94.6±6.7	93.1±78.8	91.4±7.0	91.8±8.8	91.9±7.5
HOMA-IR	0.9±0.7	0.9±0.7	0.8±0.5	1.0±1.0	1.1±1.2	1.1±1.3

Data are presented as mean ± standard deviation. *p*-values < 0.05 were regarded as statistically significant. Mean differences were assessed between visits separately in both groups (control group and intervention group) by using a linear mixed model with random intercept based on the varying influence of the different study participants. Control group and intervention group were compared at each time point according to distribution by using t-test or Wilcoxon- signed ranked test. Labeled means in a row with a common superscript letter do not differ, $p < 0.05$. hsCRP, high-sensitivity C-reactive protein; V1, visit at baseline; V2, visit after four weeks of intervention; V3, visit after twelve weeks of intervention.

Table 7. Cardiovascular function of participants without cardiometabolic risk

	Control group			Intervention group		
	V1	V2	V3	V1	V2	V3
n	21 (8m, 13f)			37 (17m, 20f)		
**Blood pressure**						
Systolic blood pressure, mmHg	122±14.2[a]	116±13.3[b]	121±18.9[a]	129±12.4*	128±14.9*	128±15.0
Diastolic blood pressure, mmHg	77±7.7[a]	74±8.5[b]	75±9.8[a,b]	82±8.1	82±10.2*	82±8.6*
Heart rate, beats/minute	58±8.0[a]	60±8.9[b]	56±7.7[a,b]	59±7.9	60±10.6	58±8.4
**Intima media thickness**						
Arteria right, mm	0.6±0.1	NA	0.7±0.1	0.7±0.1	NA	0.7±0.1
Arteria left, mm	0.6±0.1	NA	0.7±0.1	0.7±0.1	NA	0.7±0.1
**Pulse wave velocity**						
Augmentation index aortic, %	37.3±14.3	35.5±12.7	36.9±12.9	36.6±14.1	37.21±14.8	38.9±13.5
Central systolic blood pressure, mmHg	110±15.8	111±13.9	111.3±16.0	119±16.2	118±15.9	117.8±14.5
Pulse wave velocity, m/s	8.0±1.1	8.2±1.3	7.7±1.1	8.4±2.0	8.3±1.8	8.5±2.1

Data are presented as mean ± standard deviation. *p*-values < 0.05 were regarded as statistically significant. Mean differences were assessed between visits separately in both groups (control group and intervention group) by using a linear mixed model with random intercept based on the varying influence of the different study participants. Control group and intervention group were compared at each time point according to distribution by using t-test or Wilcoxon- signed ranked test. Labeled means in a row with a common superscript letter do not differ, $p < 0.05$. V1, visit at baseline; V2, visit after four weeks of intervention; V3, visit after twelve weeks of intervention. *, different from control, $p < 0.05$.

Table 8. Baseline and follow- up parameters during the 12-week intervention period in both intervention and control group.

	Control group		Intervention group	
	V1	V3	V1	V3
n	11 (5m, 6f)		24 (12m, 12f)	
Zonulin, ng/ml	18.4±4.7	19.6±4.1	18.2±3.6	17.4±3.2
LBP, mg/dl	0.84±0.26	0.88±0.32	0.69±0.20	0.73±0.22
Sucrose, % urine recovery	0.06±0.03	0.11±0.06	0.11±0.07	0.09±0.06
Mannitol, % urine recovery	9.05±4.22	12.18±5.33	11.73±2.88	10.55±3.6
Lactulose, % urine recovery	0.17±0.07	0.23±0.09	0.23±0.08	0.22±0.11
Lactulose/mannitol ratio	0.02±0.02	0.02±0.01	0.02±0.01	0.02±0.01
Sucralose, % urine recovery	0.91±0.94	1.22±1.02	0.71±0.35	0.88±0.58

Data are presented as mean ± standard deviation. According to distribution mean differences were assessed by using t-test or Wilcoxon- signed ranked test. *p*-values < 0.05 were regarded as statistically significant. Labeled means in a row with a common superscript letter do not differ, $p < 0.05$. LBP, lipopolysaccharide binding protein; V1, visit at baseline; V3, visit after twelve weeks after intervention.
