# Supplementary material for: Offering Fiber-Enriched Foods Increases Fiber Intake in Adults With or Without Cardiometabolic Risk: A Randomized Controlled Trial
Source: Front Nutr. 2022 Feb 16;9:816299. doi: 10.3389/fnut.2022.816299 (PMC8890034; doi:10.3389/fnut.2022.816299)
Supplement: Supplementary file 2 [file Data_Sheet_2.pdf]

Supplementary Figures

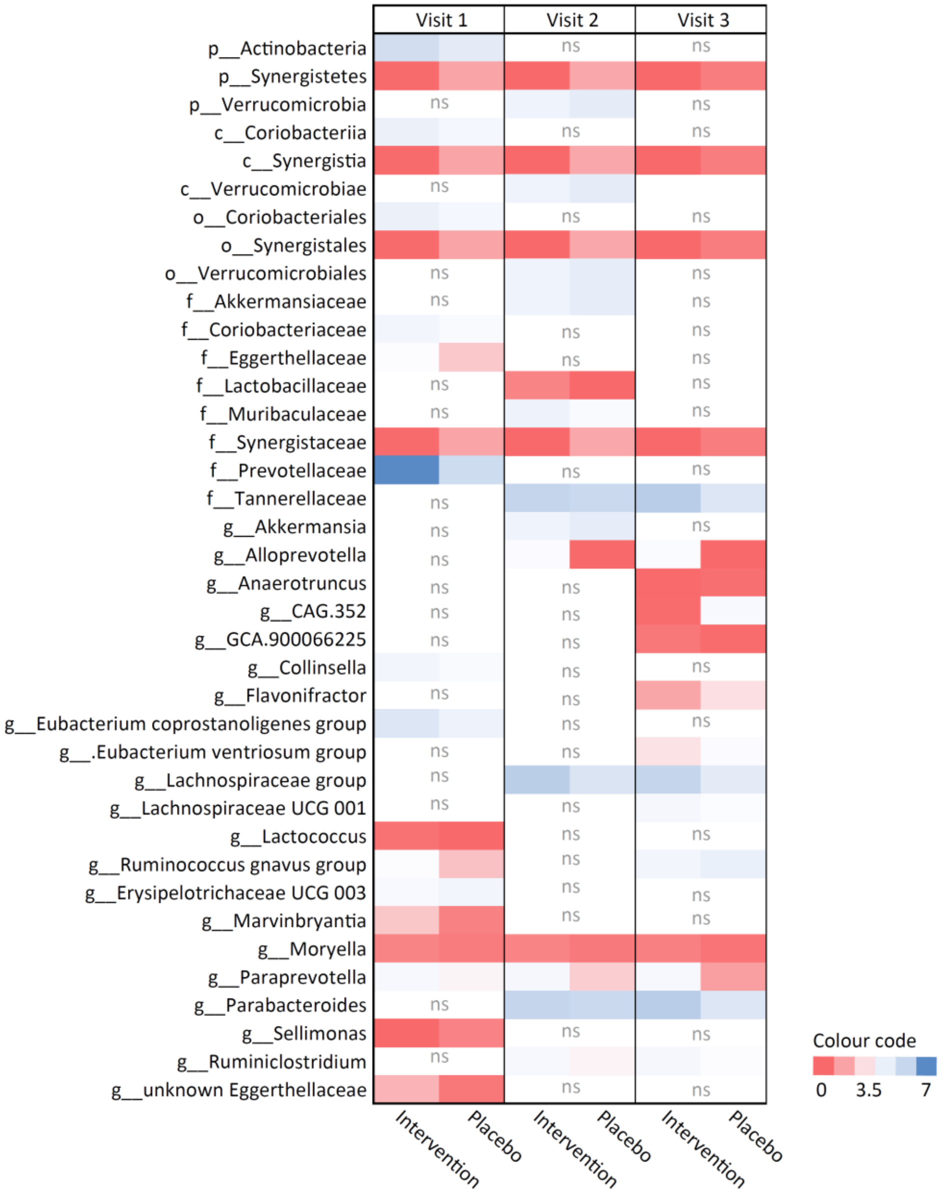

Supplementary Figure 1. Heatmap with significant different taxonomies between intervention and placebo group over time. The heatmap shows the relative abundance values of significant different taxonomies (Kruskal-Walis Test ≤ 0.05; pairwise comparison Wilcox-Rank-Sum Test ≤ 0.05). Low abundant taxonomies are shown in red and high relative abundance values in blue (see legend for colour code). Taxonomies where no significant was observed are labelled as ‘ns’. Samples are grouped according to assigned group (intervention vs. placebo group) and to Visit.

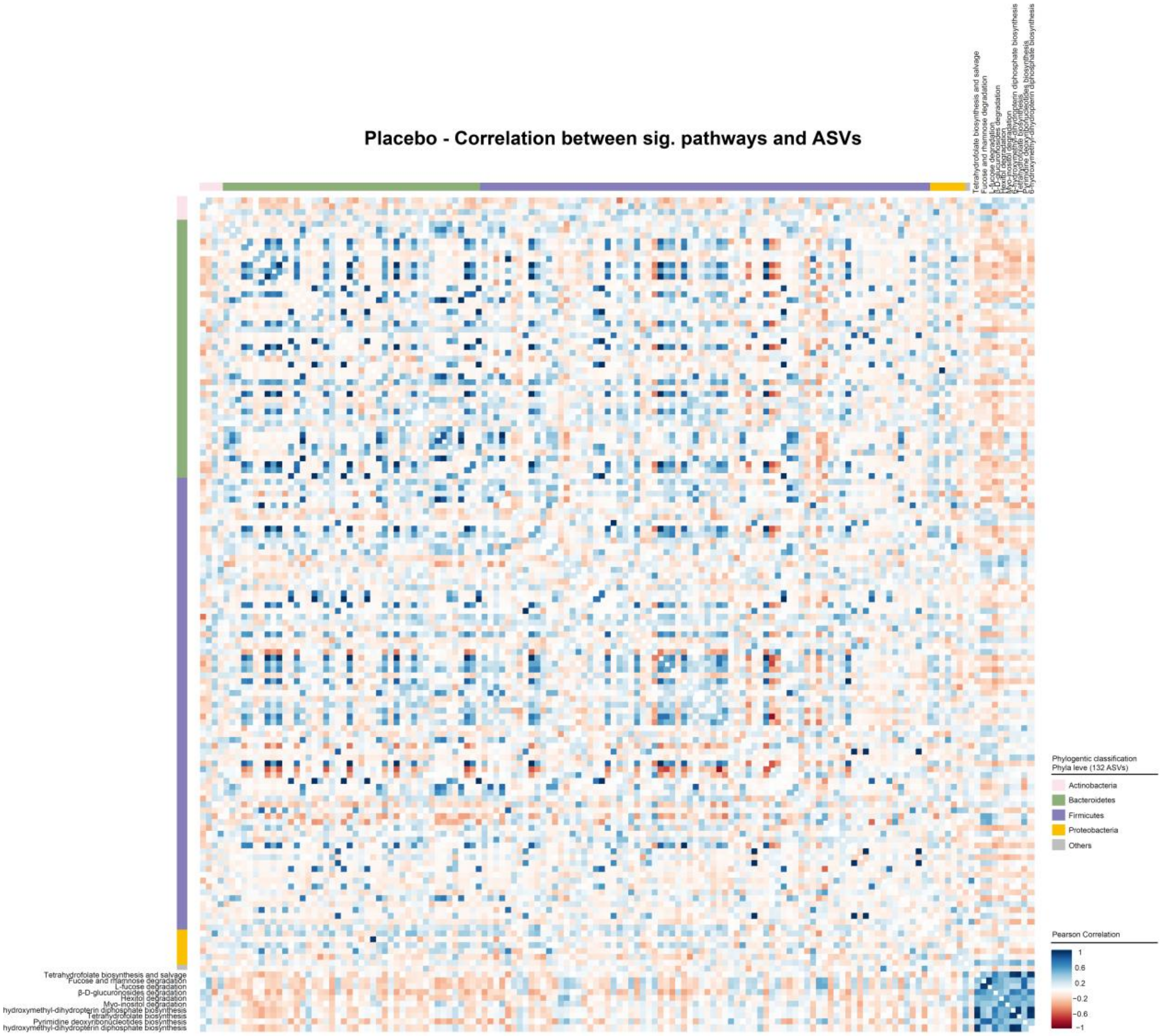

Supplementary Figure 2. Predicted functional pathways (PiCRUST2) which are significantly different between Visits in the placebo group. Heatmap shows significant correlation between selected pathways (Pearson's R ≥ 0.3; Pearsons's R ≤ -0.3) and ASVs. Strong negative correlations are shown in red and positive correlation are shown in blue. Taxonomic classification based on phyla level of the correlating ASVs is shown by colour-coded bar on the left side/top of the heatmap.
